# Polypoidal choroidal vasculopathy as a complication of choroidal osteoma: A case report

**DOI:** 10.1097/MD.0000000000019927

**Published:** 2020-05-15

**Authors:** Doyeon Kim, Gahyung Ryu, Min Sagong

**Affiliations:** Department of Ophthalmology, Yeungnam University College of Medicine, Daegu, South Korea.

**Keywords:** bevacizumab, choroidal neovascularization, choroidal osteoma, optical coherence tomography angiography, polypoidal choroidal vasculopathy

## Abstract

**Introduction:**

Choroidal osteoma (CO) is a rare benign tumor that particularly affects young, healthy women. Its prognosis is influenced by complications, such as choroidal neovascularization (CNV), subretinal hemorrhage, subretinal fluid (SF), decalcification status, and overlying retinal pigment epithelium (RPE) atrophy. In case of CNV as the complication of CO, it is typically present in the classic form; however, reports on polypoidal choroidal vasculopathy (PCV) have been rare. Here, we report a case of an older, male patient with PCV as a complication of CO.

**Patient concerns:**

A 70-year-old male patient visited the hospital with vision impairment in the right eye since 2 weeks.

**Diagnosis:**

Fundus examination revealed a red-yellow, well-demarcated, scalloped lesion around the optic nerve in each eye; the lesions were highly reflective on ultrasound examination, and thus, CO was diagnosed. Indocyanine green fluorescence angiography and optical coherence tomography (OCT) revealed that the right eye also had PCV accompanied with SF. OCT confirmed the presence of large quiescent type 1 CNV bilaterally in decalcified areas of the lesions adjacent to the optic nerve.

**Interventions:**

Intravitreal bevacizumab (IB) injection was performed.

**Outcomes:**

Best-corrected visual acuity had improved and OCT showed a decrease in the SF, while OCT angiography showed partial regression of branching vascular network.

**Conclusion:**

CO can be accompanied by quiescent type 1 CNV; this should be closely monitored because it can progress to PCV. Optical coherence tomography, alongside indocyanine green fluorescence angiography, is useful for the diagnosis and monitoring of potential CNV as a complication of CO.

## 1 Introduction

Choroidal osteoma (CO) is a rare benign tumor occurring in mostly young, healthy females.^[1,2,3,4]^ Approximately 80% of these cases are unilateral, but bilateral CO cases have also been observed, and the time of onset can differ between the eyes.^[4,5]^ Tumors are usually well-demarcated, white-yellow, or red-yellow lesions around the optic disc and can be diagnosed by characteristic fundus findings and ultrasonography. In 8% to 30% of the patients, CO is asymptomatic and is discovered by chance, but it can also present with symptoms, such as reduced visual acuity, metamorphopsia, and visual field defects.^[2,4]^ Visual prognosis is affected by the presence or absence of complications, such as choroidal neovascularization (CNV), subretinal hemorrhage, subretinal fluid (SF), decalcification status, and overlying retinal pigment epithelium (RPE) atrophy; of these, CNV is known to be a major cause of visual impairment.^[2,3,4]^ In cases of CNV as a complication of CO, CNV is typically of the classic type^[2,4,5]^; however, reports of cases with polypoidal choroidal vasculopathy (PCV) have been rare.

Here, we report our experience of a case of PCV in an older male patient with CO and present a multimodal imaging analysis, including optical coherence tomography angiography (OCTA).

## 2 Case presentation

A 70-year-old male patient visited the hospital with the complaint of visual impairment in his right eye that had started 2 weeks previously. The patient had a history of coronary artery stent insertion for acute myocardial infarction 20 years ago and had been taking medication for diabetes and hypertension for the past 19 years. He had no other ophthalmological or systemic history.

**Figure 1:**
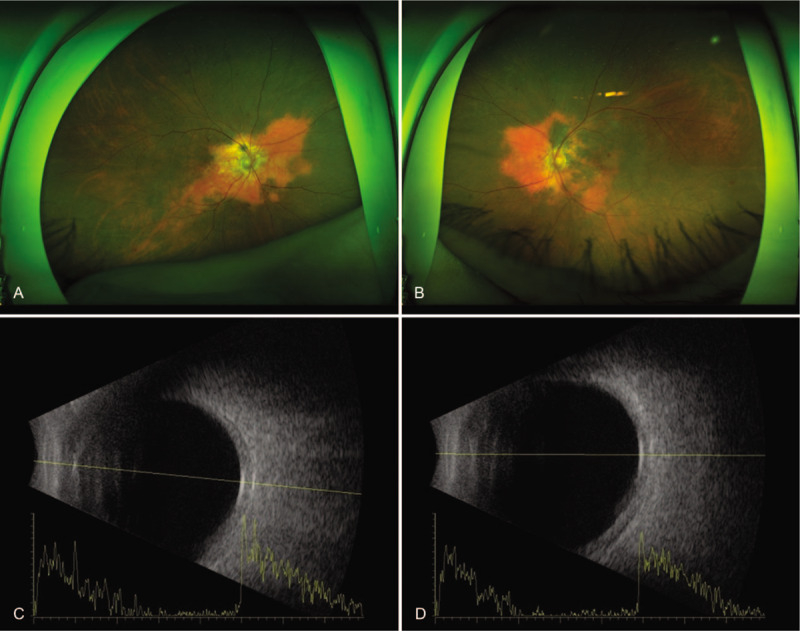
Ultra-widefield fundus photographs of the right (A) and left (B) eyes reveal well-demarcated, red-yellow colored lesion at the peripapillary area involving decalcification (yellow–white colored area). B-scan ultrasonography of the right (C) and left (D) eyes demonstrate a highly reflective choroidal mass, indicating bilateral choroidal osteomas.

**Figure 2:**
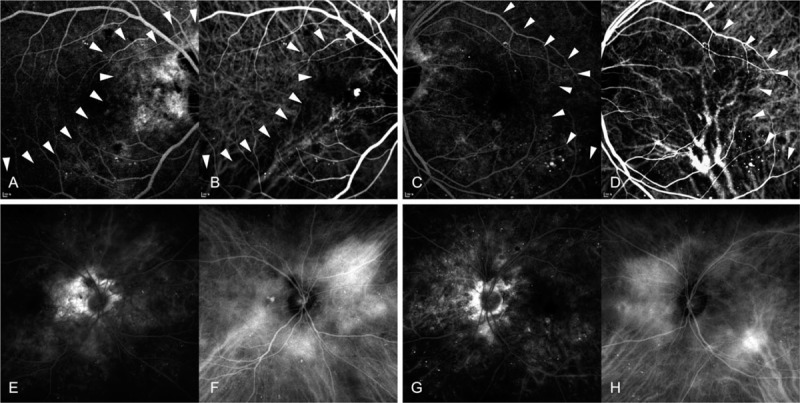
Early (A–D) and late (E–H) phase fluorescein angiography and indocyanine green angiography (ICGA) of both the eyes. On the fluorescein angiography, early patch hyperfluorescent filling pattern (A, C) with late diffuse staining (E, G) corresponding tumor lesion (arrow head) was demonstrated in both the eyes. The ICGA revealed early hypofluorescence (B, D), followed by late confluent hyperfluorescence (F, H) in both the eyes. ICGA of the right eye confirmed the presence of a polypoidal lesion with branch vascular network, indicating polypoidal choroidal vasculopathy.

**Figure 3:**
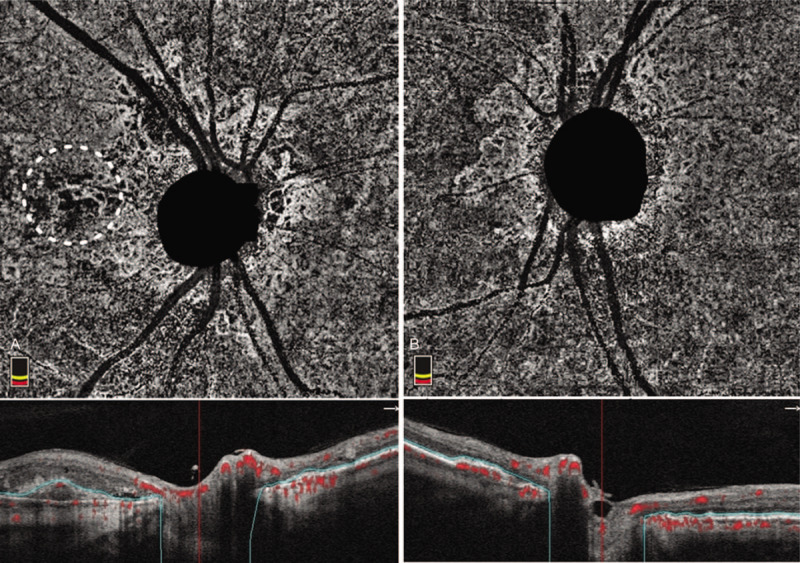
Peripapillary optical coherence tomography (OCT) angiography of the right (A) and left (B) eyes reveal quiescent type 1 choroidal neovascularization (CNV) located beneath the irregular retinal pigment epithelium (RPE) layer that is circumferentially located around the disc. In the area temporal to the disc of the right eye, a hypoflow round polyp and a hyperflow branch vascular network (dotted circle), extending from the peripapillary quiescent CNV, were observed on OCT angiography. A horizontal OCT B-scan showed turbid subretinal fluid accumulation at the corresponding area of the right eye.

At the time of the visit, the patient's best-corrected visual acuity was 0.6 in the right eye and 0.8 in the left eye, and in slit-lamp examination, neither eye showed any abnormal findings in the anterior segment. In fundus examination, both the eyes showed a red-yellow, well-demarcated, scalloped lesion around the optic nerve, which was accompanied by moderate non-proliferative diabetic retinopathy, including microaneurysms and dot hemorrhages spanning all 4 quadrants (Fig. 1A and B). On B-scan ultrasonography, the lesions around the optic nerve were highly reflective, and thus, the patient was diagnosed with bilateral CO (Fig. 1C and D). Fluorescein angiography showed hyperfluorescence in the early phase and diffuse staining in the late phase, while indocyanine green angiography showed hypofluorescence in the early phase and hyperfluorescence in the late phase; these findings are consistent with CO (Fig. 2). In peripapillary optical coherence tomography (OCT), irregular RPE elevation was observed in some decalcified areas of the lesions, and OCTA confirmed the presence of CNV below these regions. Macular OCT showed SF involving the macula, together with polyps and a characteristic double-layer sign on the nasal side. Moreover, indocyanine green fluorescence angiography revealed an abnormal branching vascular network (BVN) and polypoidal lesion that originated from the BVN; based on these findings, PCV was diagnosed. OCTA revealed the BVN as a hyperflow lesion, while the polypoidal lesion was a round structure with hypoflow (Fig. 3).

**Figure 4:**
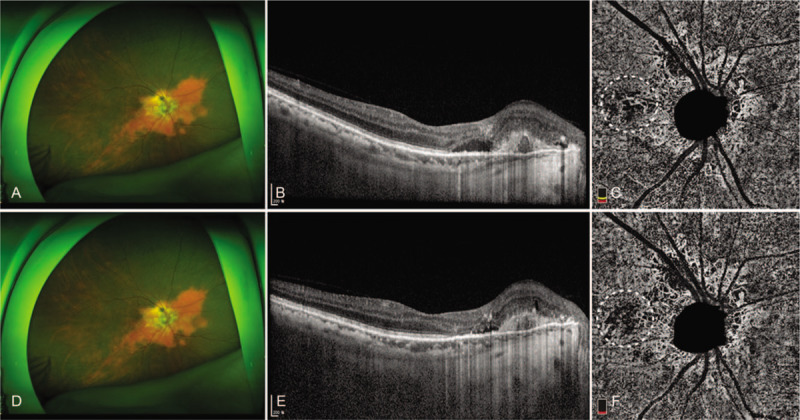
Multimodal images of the right eye at the initial examination (A–C) and 1 month after intravitreal bevacizumab injection (D–F). At the initial examination, an optical coherence tomography (OCT) B-scan demonstrated subretinal fluid accumulation with a polyp and OCT angiography shows the polyp with a hypoflow round structure and hyperflow branching vascular network (dotted circle) extending from the quiescent peripapillary choroidal neovascularization. Note the decreased subretinal fluid (E) and partial regression of BVN 1 month after intravitreal bevacizumab injection.

Intravitreal bevacizumab (IB) (Avastin; Genetech Inc., San Francisco, CA) 1.25 mg (0.05 cc) was injected into his right eye to treat the accompanying PCV. One month after a single IB injection, his best-corrected visual acuity had improved to 0.8, and OCT showed a decrease in the SF, while OCTA showed partial regression of BVN (Fig. 4).

## 3 Discussion and conclusions

CO is a rare benign tumor consisting of mature bone; however, the exact cause and pathogenesis remain unknown. It mostly occurs unilaterally in young, healthy women, but 33% of cases occur in males and 21% of cases are bilateral, as in our patient. The mean age at initial examination is 26 years, but cases have been reported in patients as young as 4 weeks and as old as 67 years.^[3]^ At initial examination, 62% of CO patients have good visual acuity of at least 0.5, but 56% to 58% show a decrease in visual acuity to 0.1 or less after 10 years; this visual prognosis varies depending on the tumor location, and complications, such as decalcification, RPE atrophy, and CNV.^[3,4]^ In cases of sudden decline in visual acuity, CNV and serous retinal detachment can be considered. CNV occurs in 31% to 47% of patients within 10 years of CO onset, and in 46% to 56% of patients within 20 years.^[3,4]^

The mechanisms of CNV occurrence in patients with CO have not been elucidated. Shields et al claimed that RPE and Bruch membrane disruption in the course of decalcification allow the growth of new underlying choroidal vessels.^[2]^ Decalcification refers to a process involving choroid atrophy, RPE changes, and photoreceptor loss as the osteoclastic component of CO causes bone resorption. It occurs in 50% of patients with CO within 10 years after onset.^[6]^ Foster et al observed osteoclasts in histological examination of CNV tissue of a patient with CO and claimed that CNV may represent an extension of the osteoma.^[7]^ Supporting this claim, 1 previous case report showed neovascularization at the center of the CO, with no decalcification.^[8]^ In our case, we observed irregular RPE elevation bilaterally throughout the regions of decalcification around the optic nerve and performed OCTA to confirm extensive type 1 occult CNV in these areas. This implies that as a complication of CO, quiescent type 1 CNV can be accompanied, and depending on the course of the disease, it can progress to PCV with the appearance of aneurysmal type 1 CNV.

Most reported cases of secondary CNV in CO involve the type 2 classic CNV, and only 1 case of PCV as a complication of CO has been reported.^[3,4,7,15]^ This may be because most studies of CO were published before description of PCV in the literature and because CO is a rare disease, large-scale studies have not yet been conducted enough to fully understand the pathogenesis and prognosis of the disease.^[3]^ PCV is known to differ from classic CNV in its course and prognosis after treatment. Therefore, it is clinically important to differentiate the 2 conditions.^[9]^

There are no known treatment methods for CO per se; however, there have been attempts to treat secondary CNV in patients with CO by surgical removal, laser photocoagulation therapy, transpupillary thermotherapy, and photodynamic therapy; however, these have shown limited effectiveness.^[7,10,11,12]^ Because anti-vascular endothelial growth factor agents have been accepted as the most effective method for treating CNV, they have also been used to treat patients with CNV accompanying CO, and several case reports have shown acceptable efficacy in improving retinal structure and visual acuity.^[13,14]^ There is 1 earlier case report of PCV as a complication of CO, in which the patient's vision, retinal hemorrhage, and SF were improved after photodynamic therapy,^[15]^ but no other treatment methods have been reported thereafter. In our case, we administered IB to treat CO accompanied by PCV and observed anatomical improvement and recovery of visual acuity after the treatment.

CNV is known to occur as a complication in approximately one-third of patients with CO.^[3,4]^ In addition to classic CNV, the possibility of PCV, which can be progressed from quiescent type 1 occult CNV, as a complication must be considered, and intravitreal anti-vascular endothelial growth factor injection may be a potential treatment. Used alongside angiography, OCTA can help with diagnosis and monitoring of CNV in patients with CO.

## Author contributions

**Data curation:** Gahyung Ryu.

**Investigation:** Min Sagong.

**Supervision:** Min Sagong.

**Validation:** Gahyung Ryu.

**Writing – original draft:** Doyeon Kim.

**Writing – review & editing:** Min Sagong.
